# A Survey of Preferences for Sensing Technologies in People with Severe Mental Illness Admitted to an Acute Psychiatric Unit

**DOI:** 10.1007/s10439-025-03769-0

**Published:** 2025-06-25

**Authors:** Alvaro Barrera, Ana Ghenciulescu, Niamh Owens, Ariana Cortez, Riccardo De Giorgi, Phil Cowen, Jeroen Bergmann

**Affiliations:** 1 Department of Psychiatry, Oxford University, Oxford, OX3 7JX UK; 2 Oxford Health NHS Foundation Trust, Warneford Hospital, Oxford, OX3 7JX UK; 3 Medical Sciences Office, John Radcliffe Hospital, Headley Way, Headington, Oxford OX3 9DU UK; 4 Department of Research & Development, Oxford Health NHS Foundation Trust, Warneford Hospital, Oxford, OX3 7JX UK; 5 Department of Technology & Innovation, University of Southern Denmark, Odense, Denmark; 6 Natural Interaction Lab, Department of Engineering, University of Oxford, Oxford, UK

**Keywords:** Sensors, Body sensor systems, Innovation, Technology, Persecutory delusions, Patients’ preferences

## Abstract

**Purpose:**

This study presents the views of patients admitted to an adult acute mental health unit due to an acute episode of severe mental illness, mainly of a psychotic nature, regarding sensing technology.

**Methods:**

One hundred and twenty five adult inpatients were approached; 31 patients declined to participate whilst eleven patients were missed due to having been discharged from hospital or transferred to a different unit. Eighty three patients consented to participate and were administered a survey, previously validated in people with arthritis and other physical health conditions, about their preferences regarding sensing technology. The participants’ clinical presentation was characterised using standard clinical instruments.

**Results:**

Ninety percent of participants were on antipsychotic medication and 78% were detained in hospital under the Mental Health Act 1983 of England and Wales. 67.9% exhibited elevated levels of ideas of reference and 61.7% presented a clinical level of persecutory delusions. Patients’ views about wearable digital technology were broadly positive and similar to those previously provided by people with arthritis and physical health conditions, with some specific differences. For example, they agreed to wear a device that is visible to others but less so to wearing one that is concealed in their clothing or implanted or for 24 h monitoring with the data being sent away, analysed or stored.

**Conclusion:**

This study shows that those who are in the middle of an acute and severe episode of mental ill health are open to using sensing technology, with some specific requirements that would increase the uptake within this patient population. This research shows that this currently underserved patient population has a positive view with regards to sensing technology for healthcare purposes. These findings can inform the design of new wearable systems, which can address the unmet needs in this clinical domain.

## Introduction

People with severe mental illness (SMI) face numerous medical, psychological, and social challenges that are associated with increased morbidity and mortality [1, 2]. Sensing technologies, and in particular wearable medical sensors, can empower people to gain ownership of their health by consistently providing information on relevant risk factors and determinants of illness that change over time [3]. These data can foster collaborative work and shared decision-making between patients and clinicians, leading to improved outcomes [4]. The work reported here focuses on patients who were acutely unwell, in hospital, most suffering from an acute psychotic episode.

Sensing technology offers several advantages for capturing clinically actionable information, such as convenience, cost-effectiveness, and the possibility to obtain data in real-world settings [5]. A range of wearable devices have been developed for monitoring patients with diagnoses of depressive and anxiety disorders. Two recent reviews focussed on wearable sensors looking at anxiety disorder and panic attacks: they retrieved a vast array of ‘off-the-shelf’ wearable devices measuring physiological data (e.g., heart rate, sleep and breathing patterns), which could potentially be used to identify and monitor anxiety and depression in real-time [6, 7]. These devices could be worn on the wrist, chest, or other parts of the body, and often synchronised with a smartphone or another device to provide real-time data and analysis thereof [6, 7]. An exploratory analysis of data from the Health Information National Trends (HINTS) survey, a nationally representative cross-sectional probability survey administered to US public data, collected between 2019 and 2020 and responded by 9303 individuals of which a minority had had cancer or heart or lung diseases, showed that using wearable devices to monitor physical health led to better self-care ability, health perception, workout duration, and body mass index (BMI) changes, which in turn were associated with a reduction of psychological distress measured as a combination of depression and anxiety items [8]. Further, a qualitative study of young adults with mood disorders found that wearables and mobile apps were considered acceptable and feasible to detect mental health deterioration in real-time by monitoring changes in sleep pattern, mood or activity levels [9]. Notably, patients in the latter study were stable in their mental status and were not suffering from an acute mental health crisis, so their views were unlikely to significantly differ from those of the general population.

While more evidence is being gathered in anxiety and depression, the views around sensing technology of those going through a severe episode of mental ill health remain unclear and possibly associated with higher prejudice and stigma. Crucially, these patients’ perspectives might significantly differ from the general population due to the influence of persecutory ideation, but this has not been formally assessed. Intriguingly, a survey completed by 215 people with a diagnosis of schizophrenia spectrum disorder, showed an increase in ownership of smartphones, which could facilitate the use of apps for therapeutic interventions; however, there was a sub-group of participants whose paranoid ideation represented a barrier to owning a smartphone [10]. Another study of a wearable device worn on the participant’s wrist in 28 outpatients with schizophrenia and 25 control subjects, found similar levels of acceptability of around 80% in both groups [11]. The literature thus far covers patients who are not dealing with a severe mental health crisis, so the issue of acceptability of devices developed for people with severe mental illness remains unclear [12] yet crucial as it is at this crossroad that real-time monitoring may provide information about relapse of illness.

To our knowledge, there is no information as to whether patients with an acute episode of their severe mental health condition, in particular when suffering from an acute psychotic episode requiring hospitalisation, would reject using a wearable monitoring system. Similarly, it is not known whether those views would be different from those of people with other significant physical health conditions. It is therefore important to establish whether patients facing an acute episode of severe mental illness would accept sensing technology and, if they do, whether and how their views on this matter might differ from other patient groups or the general population. A recent study that used a wearable biosensor in young psychiatric inpatients with autism spectrum disorders reported that only 8% of the patients did not want to wear the provided device during the research trial [13]. A better understanding of these issues can facilitate the development of new sensors applications; conversely, these systems will fail if there is a lack of user compliance and might even worsen the mental health of a patient if their preferences are not duly considered.

This study is the first investigation that aims to collect information regarding monitoring preferences of sensor systems from inpatients (i.e., patients admitted to an adult acute mental health unit), who are dealing with an acute mental health crisis, using a survey approach. This survey mirrors the method of a prior study that surveyed (mainly) osteoarthritis patients [14], where the questionnaire was assessed for face validity and test–retest reliability, with dichotomous items showing an agreement of 95%, whilst 3-point Likert-type scale questions yielded a moderate Kappa of 0.55 across all questions. Using this questionnaire, we compared preferences for wearable sensors between these two groups of patients. In addition, given its relevance to the acceptability of monitoring devices, information was gathered with regards to paranoid beliefs.

## Materials and Methods

### Recruitment Strategy

Recruitment involved patients admitted to the adult acute mental health units, at the Warneford Hospital, Oxford Health NHS Foundation Trust, Oxford, UK. The survey was presented to the respective wards’ consultant psychiatrists, who then signposted potential participants to the study team after checking inclusion and exclusion criteria - see below. Patients were then approached by the study team and provided with the Patient Information Sheet (PIS). After reading it and being given the opportunity to ask any questions, they had several days to decide whether to participate in the study. Interested patients were then approached again to establish whether they had capacity to consent to participate in the study; if they accepted to take part, written informed consent was obtained, and a study laptop was provided for accessing the form. All patients were offered the option to complete the online form independently with a team member available to clarify any concerns, or to receive more support with filling it out. Some participants preferred for the questions to be read out to them – common reasons for this choice included self-reported problems with attention, reading speed or comprehension, or not feeling comfortable with the technology. All interviews took place in person in the inpatient wards at the Warneford hospital.

### Eligibility Criteria

All patients had to be aged 18 to 65 and had been hospitalised, with an International Classification of Diseases (ICD-10) [15] diagnosis of severe mental illness including the following illnesses and diagnostic codes: schizophrenia (F20), delusional disorder (F22), acute and transient psychotic disorder (F23), schizoaffective disorder (F25), other nonorganic psychotic disorders (F28 and F29), manic episode (F30) or bipolar affective disorder (F30, F31), severe depressive episode with psychotic symptoms or recurrent depressive disorder current episode severe with psychotic symptoms (F32.3, F33.3).

Patients who were assessed as lacking capacity to consent to the study were excluded.

### Study Procedures

The Patients Preference on Sensors Questionnaire (PPSQ) was originally designed and validated [14] as a structured online set of both open-ended and closed questions aiming to comprehensively capture the views of healthcare users regarding wearable and implantable sensing devices. Specifically, the combination of both free-text boxes (e.g., “What should the device look like?”) and pre-compiled options (e.g., “Would you wear a device that is visible to others?”, Yes/No) allows participants to provide thoughtful responses on health sensors based on their own views, being encouraged to think beyond the limitations of current technology and imagine potentially unlimited possibilities, while also allowing us to obtain quantitative data that is directly comparable between patients. In this study, patients used a laptop to complete a bespoke version of the PPSQ (Supplementary Material), while a member of the study team was available to support participants regarding any further potential query they might have had or if they experienced any significant distress.

In order to further characterise the severity of the patients’ persecutory delusions, the second part of the questionnaire consisted of the Revised Paranoid Thoughts Scale (RGPTS) [16], an established questionnaire that assess paranoia, containing eight items related to ideas of reference (Part A) and ten items related to ideas of persecution (Part B), each scored from 0 to 4. A value higher than 10 across Part A and a value of 6 or higher for Part B would indicate high level of paranoia. A score of 11 or higher in part B would indicate clinical levels of persecutory ideation. In total, the study procedure lasted about 30 min and participants were compensated with £5 for their participation.

After completing the above survey, researchers recorded the following information for each participant: age, sex assigned at birth (male, female), primary diagnosis (schizophrenia, other psychosis, schizoaffective disorder, mania or bipolar affective disorder, depression with psychosis) and secondary diagnoses (substance use disorders, other mental health comorbidities), current medications (antipsychotics including depot and oral medications, mood stabilisers, antidepressants, hypnotics, other psychotropics, non-psychotropic medications), and Mental Health Act 1983 of England and Wales status (voluntary patient, Section 2, Section 3). Based on the patients’ electronic healthcare records, the study team completed the following rating scales to clinically characterise the sample:

- Brief Psychiatric Rating Scale (BPRS) [17],
- Young Mania Rating Scale (YMRS) [18],
- Hamilton Depression Rating Scale (HDRS) [19],
- Clinical Global Impressions Scale (CGI) [20].

Participants were de-identified with a code linking their demographics and clinical information with their responses to the PPSQ and RGPTS.

### Statistical Analysis

Participants’ demographics and clinical characteristics were reported descriptively. For the PPSQ and RGPTS, a frequency analysis was applied to the answers to all available questions. The responses to PPSQ open-ended questions were analysed using natural language processing (NLP), which consisted of converting the text to lowercase followed by stemming. All words that did not reflect the focus of the question, including stop words, were removed before the frequency analysis was performed. The responses to the PPSQ Likert scale questions were analysed for the sample. In these questions, the participants were asked on a scale from 1 to 10 how important statements were (e.g., ‘A body worn sensor device should be reliable’), with 10 being the highest. These results were then compared with those obtained in the previous study of (mainly) osteoarthritis patients [14]. In particular, we compared the frequency of the answer ‘Yes’ to questions related to the use of a wearable devices, to establish whether acceptability of acutely unwell hospitalised mental health patients is different to that of patients with physical health conditions. A Chi-square test for homogeneity was performed to determine if physical health patients and acutely unwell psychiatric inpatients had the same frequency distribution for the dichotomous (yes/no) questions related to the device preferences. This subsection of questions had previously been reported on for the physical health patients (14) and thus was available for comparison with the frequencies obtained for the acutely unwell psychiatric inpatients in this study.

### Ethics and Data Processing

The study was formally approved as a Clinical Audit and Service Evaluation Proposal by the Quality and Audit Team, Oxford Health NHS Foundation Trust (Adult MH—Acute Inpatient/SE/2024-25/04) on 7th of April 2022. Responses were entered online using a General Data Protection Regulation (GDPR) compliant, custom-made Jisc interface (Jisc, Bristol, UK). No identifiable information was entered in the online database. All information was de-identified and stored in a password-protected cloud service.

## Results

### Demographic and Clinical Characteristics of the Sample

A total of 125 inpatients who potentially met eligibility criteria were approached: 31 patients declined to participate, and 11 patients were missed due to either having been discharged from hospital or transferred to another hospital before being interviewed. Thus, 83 participants were eventually included in the study. Table 1 displays the demographic and clinical characteristics of the study sample: the average age was 41.0 (13.7) years, 35 were females and 48 males. The most common diagnoses were schizophrenia (28.9%) and psychosis NOS (28.9%). 90.4% of the patients were on at least one antipsychotic medication. Of note, 88% of the participants were detained in hospital under a Section 2 or a Section 3 of the Mental Health Act 1983 of England and Wales [21]. Their average clinical severity at the point of interview, rated according to the Clinical Global Impression Scale-Severity, was between moderately ill and markedly ill; 19.3% were severely ill. As shown in Table 1, the patients had a range of severity in terms of symptoms of psychosis (BPRS) [15], mania (YMRS) [16], and depression (HDRS) [17].

**Table 1:** Demographic and clinical characteristics of the study sample

	Study sample
N	83
Age (years)	41.0 ± 13.7
Sex assigned at birth	
Female	35 (42.2%)
Male	48 (57.8%)
Primary diagnosis (ICD-10)	
Psychosis NOS	24 (28.9%)
Schizophrenia	24 (28.9%)
Bipolar Affective Disorder	17 (20.5%)
Major Depressive Disorder with Psychosis	6 (7.2%)
Schizoaffective Disorder	12 (14.5%)
Psychiatric comorbidity	
Substance Use Disorder	3 (3.6%)
Another psychiatric comorbidity	11 (13.3%)
Psychiatric medication	
* Antipsychotic*	75 (90.4%)
- Long-acting depot injection	13 (15.7%)
- Oral	61 (73.5%)
- Long-acting depot injection + Oral	1 (1.2%)
Mood stabiliser	13 (15.6%)
Antidepressant	9 (10.8%)
Hypnotic	58 (69.9%)
Other psychiatric medication	2 (2.5%)
Non-psychiatric medication	42 (50.6%)
MHA status	
Voluntary	10 (12.0%)
Section 2	18 (21.7%)
Section 3	55 (66.3%)
Clinical scales	
BPRS	23.9 ± 10.6
YMRS	13.8 ± 10.3
HDRS	10.5 ± 6.2
CGI	4.5 ± 1.1

Values are N (%) for categorical variables or mean ± standard deviation for continuous variables. BPRS: Brief Psychiatric Rating Scale, CGI: Clinical Global Impression scale, HDRS: Hamilton Depression Rating Scale, YMRS: Young Mania Rating Scale; MHA: Mental Health Act 1983 of England and Wales, NOS: Not Otherwise Specified

### Sensor Placement Preferences

The responses to the survey will next be described in two parts: (1) answers to open questions and (2) answers to Yes/No and Likert scale type questions.

#### PPSQ: Responses to Open Questions

**Fig. 1:**
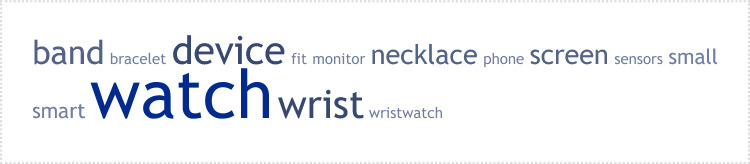
A weighted list of keywords generated for the question ‘how a wearable device should look’. The cloud provides a graphical representation of the frequency at which specific words were used by patients after all words that did not reflect the focus of the question, including stop words, were removed before the frequency analysis was performed

These responses are presented as a weighted list of words generated for the respective question, which provides a graphical representation of the frequency at which specific words were used by patients. Regarding how a wearable should look like, many participants referred back to everyday objects, rather small, discreet and unobtrusive (Figure 1). These findings are broadly similar to those reported by people with physical health conditions [14]. More specifically, 43.3% of participants mentioned that it should look like a wristwatch, 24% of participants said like a wristband or a bracelet, 9.6% of participants like a necklace, 7.2% like a smartphone, 3.6% like a ring, 2.4% like a headphone, 2.4% like glasses, 2.4% like a band around the arm, and 2.4% like other jewellery. Other options mentioned once were a tattoo, contact lenses, ankle band, around the ear, earrings, a pill to swallow, a microchip on the skin, sensors attached to the head, key chains, pendant, and stickers. Some participants mentioned more than one object/location. One patient indicated “as unintrusive as possible, very quiet. Comfortable to wear. Should have a smart watch type screen to be able to see the data.” Another patient indicated “The device should be wearable for the individual and data should be enabled to correlate with a smartphone device. Main indicators and recordings should include sleeping patterns, blood pressure, steps…the device should have an option to come with a digital screen or without. All data should be linked to a smartphone. In terms of mental health, mood should be evaluated throughout the day, i.e., using traffic light system…”

**Fig. 2:**
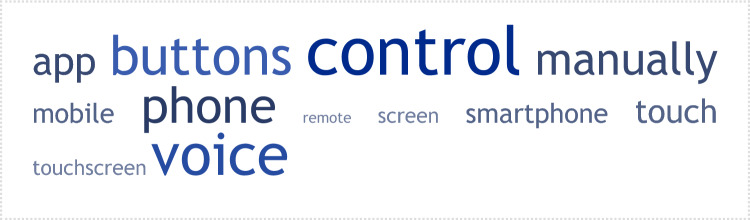
A weighted list of keywords generated for the question ‘how a wearable device should be controlled’. The cloud provides a graphical representation of the frequency at which specific words were used by patients after all words that did not reflect the focus of the question, including stop words, were removed before the frequency analysis was performed

Patients mentioned a range of ways in which the device should be controlled, including phone, voice, manually, or using an app (Figure 2). More specifically, the main options mentioned were as follow: 19.2% of participants indicated that it should be controlled by voice, 18% using a button on the device, 15.6% using a phone, 10.8% manually, 10.8% using an app, 8.4% using a touch screen, and 6% using a smartphone.

Almost all patients (96.4%) indicated that the device should be external, outside the body, and 3.6% that it should be internal, inside the body. However, 61.2% of the participants indicated that they would wear an internal device in a potential lifesaving situation but 38.75% said that they would not. Regarding which part of the body they would wear a device, the areas more frequently mentioned were the wrist (68.6%), arm (15.6%), ankle (15.6%), and neck (14.4%).

**Fig. 3:**
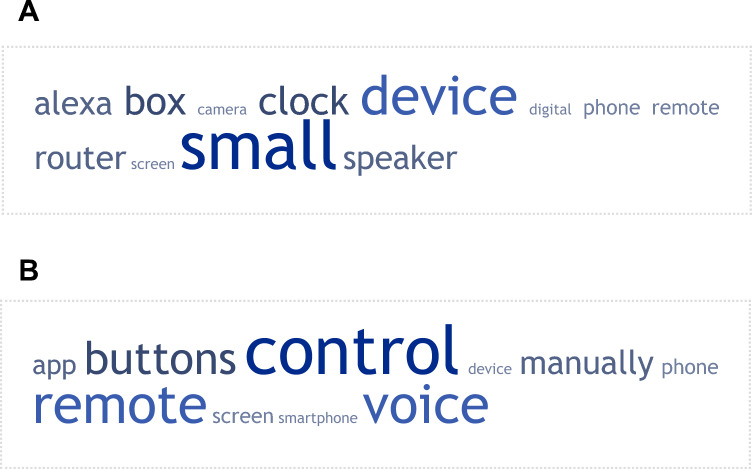
**A** and **B** A weighted list of keywords generated for the questions: ‘what the remote device should look like’ (A) and ‘how the patient should control the remote device’ (B). The cloud provides a graphical representation of the frequency at which specific words were used by patients after all words that did not reflect the focus of the question, including stop words, were removed before the frequency analysis was performed

Patients were asked their views about a remote device associated with a wearable sensor, if it was needed, specifically ‘what the remote device should look like’ and ‘how the patient should control the remote device’. The responses are presented as word cloud generated for the respective question (Figures 3A and B). Finally, when asked whether the remote device associated with a sensor should be visible, 71.1% of patients indicated that it should be visible and 25.3% of patients said that it should not be visible, with three patients not responding.

**Fig. 4:**
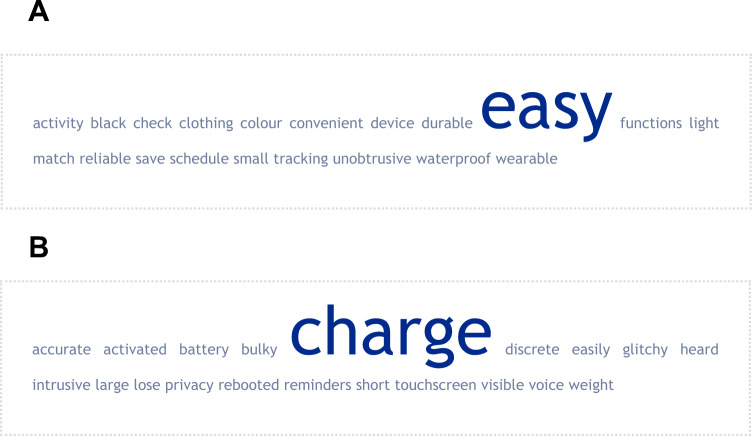
**A** and **B** A weighted list of keywords generated for the question: ‘what do you like about the device you are using’ (A) and ‘what you do not like about the device you are using’ (B). The cloud provides a graphical representation of the frequency at which specific words were used by patients after all words that did not reflect the focus of the question, including stop words, were removed before the frequency analysis was performed

When patients were asked whether they were already using a device, 14.5% indicated that that was the case, including smart/running/activity tracking watches (10), a speaker/smart device (1), and pacemaker (1). Figures 4A and B show what patients liked and did not like, respectively, about the device they were using.

#### Responses to Likert Scale Type Questions

Patients’ preferences about wearing sensing technology are shown in Table 2. A few results are notable. When asked whether they would wear a device that is visible to others, 65.1% indicated that they would and a further 20.5% that they would possibly consider it. Also, 69.9% of participants would wear it concealed in their clothes but 50.6% would not wear an implanted device. Over half (54.2%) of participants would allow continue monitoring and 61.4% would allow for the data to be sent away and analysed or stored on a database. The final question asked patients whether they would wear an implant with a sensor incorporated into it and 51.8% indicated that they would not wear such a system.

**Table 2:** Patients’ responses to Likert scale type questions regarding their preference about wearing sensing technology

Question	Yes	No	Maybe	Don’t know	Missing
Would you wear a device that is visible to others	54 (65.1%)	9 (10.8%)	17 (20.5%)	3 (3.6%)	–
Would you wear a device that is concealed in your clothing	58 (69.9%)	12 (14.5%)	10 (12.0%)	3 (3.6%)	–
Would you wear an implanted device?	17 (20.5%)	42 (50.6%)	17 (20.5%)	6 (7.2%)	1 (1.2%)
Would you allow continuous monitoring (24 h a day)	45	17	17	4	–
	(54.2%)	(20.5%)	(20.5%)	(4.8%)	
Would you allow data to be sent away and analysed or stored on a database?	51	10	20	1	1
	(61.4%)	(12.0%)	(24.1%)	(1.2%)	(1.2%)
Would you consider wearing a device to monitor how active you are?	61	10	10	1	1
	(73.5%)	(12.0%)	(12.0%)	(1.2%)	(1.2%)
Would you use a device that you had to apply yourself without assistance?	64	10	6	3	–
	(77.1%)	(12.0%)	(7.2%)	(3.6%)	
Would you like the device to determine the level of your condition (e.g., mild or severe)?	60	8	11	4	–
	(72.3%)	(9.6%)	(13.3%)	(4.8%)	
Would you wear a device that would tell the clinician if you have worn it often enough?	49	12	16	6	–
	(59.0%)	(14.5%)	(19.3%)	(7.2%)	
Would you spend time learning how to use a new wearable device?	68	8	5	2	–
	(81.9%)	(9.6%)	(6.0%)	(2.4%)	
Would you wear an implant that has a sensor incorporated in it?	20	43	14	6	–
	(24.1%)	(51.8%)	(16.9%)	(7.2%)	

The effects of age, sex assigned at birth, and clinical diagnosis on the expressed preferences were explored. There was no statistically significant difference according to sex assigned at birth (all Chi-Square [two sided] > 0.146). When divided by age (median = 40 years old), there was just one statistically significant difference on the question whether the participant would wear a device concealed in their clothing, with patients 40 or older being more likely to agree with that option (Chi-Square = 8.052; df = 3; p [two sided] = .045); this difference ceased to be significant after Bonferroni correction. There were no other significant differences regarding age. The sample was divided into two groups according to their diagnosis: one group of patients with a schizophrenia spectrum disorder (namely, schizophrenia, schizoaffective disorder and psychosis NOS) and the group of patients with an affective disorder (namely, bipolar disorder and major depressive episode). There were no significant differences between these two groups regarding their preferences (all Chi-Square [two sided] > 0.100).

Regarding how long it should take to put a sensor device on, 57.8% of patients indicated less than a minute and 25.3% of patients less than 5 min. When asked for how long they would be willing to wear such a device over a 24-h interval, 51.8% of patients said they would wear for more than 20 h, 16.9% of patients for less than 15 h, and 10.8% of patients for less than 10 h; 10.8% of patients indicated that they would wear it for less than 1 h a day. Regarding for how long the device should be able to work before the battery needed to be charged again, 21.7% of patients said it should be for more than six months, 36.1% for less than a week, 19.3% for less than a month, and 14.5% for less than a day.

Participants were subsequently asked to rate several specific features of wearable devices on a 10-point scale, with 10 being the most important (Table 3). The highest rated items here were that the device should be reliable, providing clear and useful results as well as being comfortable and simple to operate. They also endorsed the view that worn-sensors should work alongside their clinical care team instead of replacing it. They seemed to be the least concerned with the device blending in with different types of clothing or it being recyclable, although they still yielded an average importance value of 6.72 and 7.01, respectively.

**Table 3:** Participants’ ratings, on a scale of 1 to 10, on the importance of the following statements (10 being the highest)

A body worn sensor device should…	Mean (SD)	
...Be reliable	9.26	1.67
...Provide clear and useful results	9.06	1.84
...Be simple to operate (and maintain)	8.99	1.78
...Be comfortable	8.94	1.85
...Have clear and readable instructions	8.92	1.85
...Increase the accuracy of current clinical procedures	8.68	2.05
...Provide positive feedback to patients	8.63	2.27
...Work alongside your medical care team, instead of replacing them	8.55	2.45
...Not affect normal daily behaviour	8.41	2.64
...Be easy to attach to the body	8.30	2.31
...Motivate the people using it	8.28	2.34
...Give instant feedback	8.21	2.23
...Be compact (light and small)	8.20	2.41
...Speed up currently used clinical procedures	8.15	2.38
...Minimize incorrect use of the system	8.15	2.36
...Reduce travel to clinics and hospitals	8.13	2.59
...Not detach from patient unless needed	7.43	2.92
...Be discrete	7.22	2.90
...Be recyclable	7.01	3.06
...Blend in with different types of clothing to be worn with the device	6.72	2.98

### Comparing the Current Clinical Sample with a Sample of Patients with Physical Health Conditions

In order to establish whether patients hospitalised with a diagnosis of a psychotic disorder would be less likely to consider using wearable sensing technology than people suffering from physical health conditions, we compared the pattern of responses of the current sample to those given to the same PPSQ by 299 patients who self-reported some type of arthritis (52%), hypertension (12%), asthma (11%), and diabetes (10%) [14]. These physical heath patients had completed the questionnaire in an online format, whereas the current sample had it administered to them in person by a member of the study team (Table 4).

**Table 4:** Comparing the number and frequency (%) of a Yes answer to questions related to the use of a wearable device between arthritis/physical health patients [14] and the current participants

	YES response Physical health patients N: 299 (%)	YES response Acutely unwell psychiatric patients N: 83 (%)
Would you wear a device that is visible to others?	97 (32%)	54 (65%)
Would you use a device that you had to apply yourself without assistance?	240 (80%)	63 (76%)
Would you consider wearing a device to monitor how active you are?	234 (78%)	61 (73%)
Would you spend time learning how to use a new wearable device?	272 (91%)	68 (82%)
Would you like the device to determine the level of your condition (e.g., mild or severe)?	252 (84%)	60 (72%)
Would you wear an implanted device?	112 (37%)	17 (20%)
Would you allow continuous monitoring 24 h a day?	214 (72%)	45 (54%)
Would you wear a device that is concealed in your clothing?	266 (89%)	58 (70%)
Would you wear a device that would tell the clinician if you did not wear it often enough?	177 (59%)	49 (59%)
Would you allow data to be sent away and analysed or stored on a database?	249 (83%)	51 (61%)
Would you wear an implant that has a sensor incorporated in it?	156 (52%)	20 (24%)

The Chi-square test for homogeneity showed that there was a statistically significant difference (df = 10, *N* = 1413) = 32.79, *p* = .001) between the physical health patients and the acutely unwell psychiatric inpatients’ distribution of the obtained frequencies. The largest difference in frequencies (33%) was for the question “Would you wear a device that is visible to others?” The second largest difference (28%) was for the item asking if they would wear an implant that has a sensor incorporated in it. There was no difference found for “yes” responses between the two groups for the question “Would you wear a device that would tell the clinician if you did not wear it often enough?” (Table 4). We also compared the two groups regarding their endorsement of the characteristics that the body worn sensor should have (Table 5).

**Table 5:** Comparing the importance given to each statement related to the use of a body worn-sensors between arthritis/physical health patients [14] and this study’s participants

	Arthritis/physical heath patients (APHP) [14] Mean (SD)		Acute psychotic episode inpatients (APEI) Mean (SD)		Difference of means (APHP - APEI)
A body worn sensor device should…					
...Be discrete	8.5	1.9	7.2	2.9	1.3
...Minimize incorrect use of the system	9.1	1.5	8.1	2.3	1.0
...Not detach from patient unless needed	8.5	2.0	7.4	2.9	1.0
...Reduce travel to clinics and hospitals	9.1	1.6	8.1	2.5	1.0
...Speed up currently used clinical procedures	9.1	1.4	8.1	2.3	1.0
...Be compact (light and small)	9.1	1.5	8.2	2.4	0.9
...Blend in with different types of clothing to be worn with the device	7.6	2.4	6.7	2.9	0.9
...Be easy to attach to the body	9.1	1.4	8.3	2.3	0.8
...Increase the accuracy of current clinical procedures	9.3	1.3	8.6	2.0	0.7
...Be comfortable	9.6	1.2	8.9	1.8	0.7
...Not affect normal daily behaviour	9.1	1.6	8.4	2.6	0.7
...Provide positive feedback to patients	9.3	1.3	8.6	2.2	0.7
...Work alongside your medical care team, instead of replacing them	9.2	1.4	8.5	2.4	0.7
...Motivate the people using it	8.8	1.6	8.2	2.3	0.6
...Provide clear and useful results	9.5	1.2	9.0	1.8	0.5
...Be reliable	9.6	1.2	9.2	1.6	0.4
...Be simple to operate (and maintain)	9.3	1.4	8.9	1.7	0.4
...Be recyclable	7.4	2.8	7.0	3.0	0.4
...Have clear and readable instructions	9.3	1.3	8.9	1.8	0.4
...Give instant feedback	8.1	2.2	8.2	2.2	− 0.1

As shown in Table 5, both groups assigned similar importance to most items. However, there was a difference of more than one point in the mean of the following items: the patients with severe mental illness admitted to a mental health unit gave less importance to the body worn sensor device being discreet (7.2 v 8.5) and that it should minimize the incorrect use of the system (8.1 v 9.1); they gave also less importance to the body worn-sensors not detaching unless needed (7.4 v 8.5), that it reduced travel to clinics or hospitals (8.1 v 9.1), and that it should speed up current clinical procedures (8.1 v 9.1). For both groups, it was important that the body worn sensor device worked alongside their medical care team, instead of replacing them.

### Persecutory Delusions and Ideas of Reference

A total of 81 participants completed the Revised Paranoid Thoughts Scale (RGPTS) [13], with a mean Ideas of Reference total score of 12.8 (SD: 9.5) and a mean Ideas of Persecution total score of 16.2 (SD: 13.3), with 67.9% of the participants exhibiting elevated levels of ideas of reference and 61.7% presenting a clinical level of persecutory delusions. These data indicate that the patients had significantly high levels of paranoia.

## Discussion

The aim of the present study was to establish the views that patients suffering from the most severe clinical presentations in psychiatry had about using sensing technology. This is important as, relative to the general population, patients with serious mental illness have a reduced life expectancy of between 15 and 20 years. Therefore, development and implementation of interventions to reduce this mortality gap are urgently required [1, 2]. Gathering mental and physical health information in real-time is required for designing interventions that are acceptable for patients suffering from severe and enduring mental illness. The participants in this study were in the middle of a severe mental ill health crisis, involuntarily detained in hospital, on antipsychotic medication, and reporting a high level of persecutory delusions. Previous research has involved participants with anxiety or depressive disorders and [6–8] and, when involving participants with a diagnosis of bipolar disorder or schizophrenia, patients were in a rather settled and stable mental state [9–11]. To our knowledge, this is the first report investigating the views of this clinical population regarding sensing technology.

Overall, patients engaged well with the questionnaire and provided thoughtful answers. Interestingly, there was a difference in the frequency distribution across the items that explored the patient’s preferences using yes/no questions in the acutely unwell psychiatric inpatients and the physical health patients [14]. This indicates that the two patient populations do have different attitudes towards these monitoring systems. There was a marked reduction in the number of patients that wanted to wear visible devices in the acutely unwell psychiatric inpatients compared to the physical health patients. However, there were also similarities such as the willingness to have a device that would tell the clinician if they did not wear it often enough. This shows that preferences should be considered in detail to better develop suitable technologies for a particular patient group. There is an assumption for random sampling when performing the Chi-square test and the obtained sample from the hospital can be considered more of a convenience sample, as the volunteers only originated from a single hospital. We also assume that the generated contingency table consisting of physical health and acute psychotic patients was mutually exclusive and the items compared are considered not to be overlapping. Items such as wearing a device that is visible and concealed in the clothing might be considered to ask the address the same preference. However, the question of visibility yielded a difference of 33% between the two groups, whilst concealing only reached 19%. This indicates that these questions should not be considered to be addressing the same preference. Another issue to be considered when comparing the two groups, is that the current sample is composed of acute patients whilst the physical health sample is composed of patients with long term chronic conditions, a difference that could account for the found differences. In other words, the approach to the use of sensing technology could be different in acute versus chronic conditions. Overall, this information might be helpful for designing digital sensing devices that are actually used by patients with severe mental health conditions since, as the presented data suggest, they are keen on engaging with this type of technology.

Initially, less than 5% of the respondents wanted a device that would be located internally, which shows a clear preference for externally placed systems. However, the willingness to have a system inside the body increased to just over 60% if such a device was potentially lifesaving, indicating that it is important for patients to know what value a device brings, as this could influence the willingness to accept certain technologies. It should also be noted that internal devices are more than just implantable devices, as an internal device could consist of temporary measures, such as a sensor pill that could be digested or a mouthguard with embedded sensing capabilities [22, 23]. Implantable devices can be expected to be located inside the body for longer periods of time. Nonetheless, almost half of the participants were willing to have an implanted device or a smart implant, suggesting that implants could be acceptable for a large proportion of this population. Furthermore, the study can be replicated by other researchers to generate a more comprehensive understanding of how different patient populations view the use of sensor technology. Similarly, by conducting studies in other countries, cultural differences in preference could be mapped to better understand the scalability of certain approaches.

The present study has, in our view, several strengths. First, the survey was administered in person, which allowed to develop rapport and get the vast majority of participants to engage and respond all the questions. A face-to-face interview with a clinician allowed the interviewer to rephrase, assess the mental state of the patient, and/or give a further explanation within a mental health context. Moreover, a clinician is also required to assess if the patient is able to give informed consent. Second, the survey asked open questions, giving participants the option of thinking aloud about the topic without being limited to the options provided or to current examples of technology. The way in which the survey was administered tried to minimise potential concerns that arise when instruments are applied to vulnerable populations [24–26]. Thus, patients were offered the option to complete the form independently with a team member available to clarify any concerns, or to receive more support with filling it out, and some participants preferred for the questions to be read out to them, because of problems with attention, reading speed or comprehension, or not feeling comfortable with the technology. The duration of the interview was kept below 30 min to avoid fatigue.

The present study also has several limitations. First, a bigger sample size would make its findings more representative; unfortunately, we missed some patients who had initially agreed to participate due to them having been discharged from hospital or transferred to a different hospital. Second, we do not have data with regard to those patients who did not want to participate so we cannot ascertain their reasons for that decision. Third, explicitly including people from ethnic minority backgrounds as well as patients whose first language is not English should be part of further exploration. Related to the previous point, the data reported here could be specific to the UK inpatient setting and therefore more research is needed in other cultural and health care setting to determine their generalisability. Fourth, there were no significant differences in terms of age, sex assigned at birth, and clinical diagnosis on the expressed preferences about wearing sensing technology, after correcting for multiple comparisons. However, future work in this area should also consider self-reported gender to see whether it is more indicative of wearable device usage behaviour than sex assigned at birth. Fifth, we have compared our data with data from arthritis patients obtained 12 years ago and it is likely that people’s preferences regarding sensing technology may have changed due to its increasing availability over the last decade. Sixth, the current approach could be supplemented with a qualitative approach, where focus groups with the same patient population and their relatives could provide additional information which the current approach may have missed. Seventh, further research should explore whether patients’ acceptance of wearable or implantable devices differs during acute hospitalization as opposed to after discharge, which would require a longitudinal approach with follow up of patients after discharge. Penultimately, we acknowledge that the Patients Preference on Sensors Questionnaire (PPSQ) includes terms such as ‘reliable’, which have specific technical definitions in the context of wearable devices. We did not explain the technical meaning of these terms to the participants because we were interested in their perception’s as potential users of devices. Finally, whilst it could be argued that a more detailed explanation of the benefits of sensing technologies to participants (perhaps supplemented with visual aids and videos) could make their responses to reflect more accurately their perspectives, the present questionnaire aims to capture the current willingness of acutely unwell psychiatric patients to wear sensor systems without biasing them by providing additional information. This is a very vulnerable population and in practice a balance is needed between seeking information and providing suggestions on possible available sensor systems, which could have negative consequences.

How could our findings contribute to the development of more effective sensor-based mental health solutions? We have asked broad questions to a sample of acutely unwell patients who have a high degree of persecutory delusions. They have provided insights that can be of use to both the biomedical engineering community as well as mental health practitioners. For example, current sensor systems such as smart watches and devices controlled by voice, manually or remotely, are likely to be accepted so more of these available solutions should be tested within psychiatry.

The adoption of sensing technology which is acceptable to patients with severe mental illness (SMI) during acutely unwell periods as well as during more settled times, would provide mental health care providers with access to actionable data which would inform acute as well as long term care. People with SMI face numerous medical, psychological, and social challenges that are associated with increased morbidity and mortality [1, 2]. Gathering real-time physiological data with sensing technology would help to plan and potentially anticipate exacerbation of psychotic symptoms using, for example, Heart Rate Variability (HRV) obtained from a wearable device worn on the wrist [11] or quantification of negative symptoms severity using mobile phone global positional satellite (GPS) sensors [27]. Similarly, in people with bipolar affective disorder, a comfortable t-shirt with embedded sensors, such as textile electrodes, can monitor HRV, respiration activity, and activity recognition with accelerometers [28]. The impact of these and other data (e.g., sleep, EEG, skin temperature and conductance, electromyography, pulse, and blood pressure) on clinical decision-making would be significant.

In conclusion, the present data clearly show that patients who are in the middle of a severe and acute mental ill health crisis are open to using sensing technology. Their willingness to engage with such devices was rather similar to what has been reported by people with physical health conditions such as arthritis, high blood pressure and diabetes mellitus. These findings should inform the design of wearable systems that could assist in meeting the physical and mental health needs of this specific patient population.

## References

[1] https://www.gov.uk/government/publications/severe-mental-illness-smi-physical-health-inequalities/severe-mental-illness-and-physical-health-inequalities-briefing#:~:text=It%20is%20recognised%20that%20individuals,in%20unemployment%20%5Bfootnote%2020%5D

[2] Correll, C. U., M. Solmi, G. Croatto, L. K. Schneider, S. C. Rohani-Montez, L. Fairley, N. Smith, I. Bitter, P. Gorwood, H. Taipale, and J. Tiihonen. Mortality in people with schizophrenia: a systematic review and meta-analysis of relative risk and aggravating or attenuating factors. *World Psychiatry.* 21(2):248–271, 2022. 10.1002/wps.20994. 35524619 10.1002/wps.20994 PMC9077617

[3] Bonato, P. Wearable sensors and systems. *IEEE Eng Med Biol Magaz.* 29:25–36, 2010. 10.1109/MEMB.2010.936554 20659855

[4] Krist, A. H., S. T. Tong, R. A. Aycock, and D. R. Longo. Engaging patients in decision-making and behavior change to promote prevention. *Stud Health Technol Inform.* 240:284–302, 2017. 28972524 PMC6996004

[5] Zheng, W., Y. Yang, C. Liu, and W. Zhou. Recent advancements in sensor technologies for healthcare and biomedical applications. *Sensors.* 23(6):3218, 2023. 10.3390/s23063218. 36991927 10.3390/s23063218 PMC10055989

[6] Gomes, N., M. Pato, A. R. Lourenço, and N. Datia. A survey on wearable sensors for mental health monitoring. *Sensors.* 23:1330, 2023. 10.3390/s23031330. 36772370 10.3390/s23031330 PMC9919280

[7] Ahmed, A., S. Aziz, M. Alzubaidi, J. Schneider, S. Irshaidat, H. Abu Serhan, A. A. Abd-Alrazaq, B. Solaiman, and M. Househ. Wearable devices for anxiety & depression: a scoping review. *Comput Methods Programs Biomed Update.* 3:100095, 2023. 10.1016/j.cmpbup.2023.100095. 36743720 10.1016/j.cmpbup.2023.100095 PMC9884643

[8] Choudhury, A., and O. Asan. Impact of using wearable devices on psychological distress: analysis of the health information national trends survey. *Int J Med Inform.* 156:104612, 2021. 10.1016/j.ijmedinf.2021.104612. 34649113 10.1016/j.ijmedinf.2021.104612

[9] Dewa, L. H., M. Lavelle, K. Pickles, C. Kalorkoti, J. Jaques, S. Pappa, and P. Aylin. Young adults’ perceptions of using wearables, social media and other technologies to detect worsening mental health: a qualitative study. *PLoS One.* 14(9):e0222655, 2019. 10.1371/journal.pone.0222655. 31532786 10.1371/journal.pone.0222655 PMC6750581

[10] Eisner, E., N. Berry, and S. Bucci. Digital tools to support mental health: a survey study in psychosis. *BMC Psychiatry.* 23(1):726, 2023. 10.1186/s12888-023-05114-y.PMID:37803367;PMCID:PMC10559432. 37803367 10.1186/s12888-023-05114-y PMC10559432

[11] Cella, M., L. Okruszek, M. Lawrence, V. Zarlenga, Z. He, and T. Wykes. Using wearable technology to detect the autonomic signature of illness severity in schizophrenia. *Schizophr Res.* 195:537–542, 2018. 10.1016/j.schres.2017.09.028. 28986005 10.1016/j.schres.2017.09.028

[12] Sheikh, M., M. Qassem, and P. A. Kyriacou. Wearable, environmental, and smartphone-based passive sensing for mental health monitoring. *Front Digit Health.* 7(3):662811, 2021. 10.3389/fdgth.2021.662811.PMID:34713137;PMCID:PMC8521964. 10.3389/fdgth.2021.662811 PMC8521964 34713137

[13] Imbiriba, T., A. Demirkaya, A. Singh, D. Erdogmus, and M. S. Goodwin. Wearable biosensing to predict imminent aggressive behavior in psychiatric inpatient youths with autism. *JAMA Netw Open.* 6(12):e2348898, 2023. 10.1001/jamanetworkopen.2023.48898. 38127348 10.1001/jamanetworkopen.2023.48898 PMC10739066

[14] Bergmann, J. H., V. Chandaria, and A. McGregor. Wearable and implantable sensors: the patient’s perspective. *Sensors.* 12(12):16695–16709, 2012. 10.3390/s121216695. 23443394 10.3390/s121216695 PMC3571806

[15] World Health Organization (2016) International statistical classification of diseases and related health problems (10th ed.). https://icd.who.int/browse10/2016/en 10.2471/BLT.25.294650 PMC13531092 42683092

[16] Freeman, D., B. S. Loe, D. Kingdon, H. Startup, A. Molodynski, L. Rosebrock, P. Brown, B. Sheaves, F. Waite, and J. C. Bird. The revised Green et al., Paranoid Thoughts Scale (R-GPTS): psychometric properties, severity ranges, and clinical cut-offs. *Psychol Med.* 51(2):244–253, 2021. 10.1017/S0033291719003155. 31744588 10.1017/S0033291719003155 PMC7893506

[17] Overall, J. E., and D. R. Gorham. The brief psychiatric rating scale. *Psychol Rep*. 10:799–812, 1962.

[18] Young, R. C., J. T. Biggs, V. E. Ziegler, and D. A. Meyer. A rating scale for mania: reliability, validity and sensitivity. *Br J Psychiatry.* 133:429–435, 1978. 728692 10.1192/bjp.133.5.429

[19] Hamilton, M. Development of a rating scale for primary depressive illness. *Br J Soc Clin Psychol*. 6(4):278–96, 1967. 6080235 10.1111/j.2044-8260.1967.tb00530.x

[20] Guy, W. Clinical Global Impressions Scale (CGI) [Database record]. *APA PsycTests*. 1976. 10.1037/t48216-000.

[21] Act Mental Health Act 1983. https://www.legislation.gov.uk/ukpga/1983/20/contents

[22] Cheng, R., P. Haste, E. Levens, and J. Bergmann. Feature importance for estimating rating of perceived exertion from cardiorespiratory signals using machine learning. *Front Sports Act Living.* 24(6):1448243, 2024. 10.3389/fspor.2024.1448243. 10.3389/fspor.2024.1448243 PMC11458442 39381259

[23] Abdigazy, A., M. Arfan, LazziG, C. Sideris, A. Abramson, and Y. Khan. End-to-end design of ingestible electronics. *Nat. Electron*. 7(2):102–118, 2024.

[24] Bakkum, L., C. Paalman, A. Müller, A. van Eeghen, and C. Schuengel. Accessibility and feasibility of experience sampling methods for mental health research with people with intellectual disability: scoping of research and stakeholder views. *J Appl Res Intellect Disabil.* 37(2):e13190, 2024. 10.1111/jar.13190. 38361385 10.1111/jar.13190

[25] Bjertnaes, O., H. H. Iversen, and J. Kjollesdal. PIPEQ-OS–an instrument for on-site measurements of the experiences of inpatients at psychiatric institutions. *BMC Psychiatry.* 6(15):234, 2015. 10.1186/s12888-015-0621-8. 10.1186/s12888-015-0621-8 PMC4596307 26444263

[26] Nicolaidis, C., D. M. Raymaker, K. E. McDonald, E. M. Lund, S. Leotti, S. K. Kapp, M. Katz, L. M. Beers, C. Kripke, J. Maslak, M. Hunter, and K. Y. Zhen. Creating accessible survey instruments for use with autistic adults and people with intellectual disability: lessons learned and recommendations. *Autism Adulthood.* 2(1):61–76, 2020. 10.1089/aut.2019.0074. 32355908 10.1089/aut.2019.0074 PMC7188318

[27] Depp, C. A., J. Bashem, R. C. Moore, J. L. Holden, T. Mikhael, J. Swendsen, P. D. Harvey, and E. L. Granholm. GPS mobility as a digital biomarker of negative symptoms in schizophrenia: a case control study. *NPJ Digit Med.* 8(2):108, 2019. 10.1038/s41746-019-0182-1. 10.1038/s41746-019-0182-1 PMC6841669 31728415

[28] Lanata, G. Valenza, M. Nardelli, C. Gentili, and E. P. Scilingo. Complexity index from a personalized wearable monitoring system for assessing remission in mental health. *IEEE J Biomed Health Inform.* 19:132–9, 2015. 10.1109/JBHI.2014.2360711. 25291802 10.1109/JBHI.2014.2360711

